# The association between total precipitation and diarrhea morbidity: A multicountry study across diverse climate zones

**DOI:** 10.1097/EE9.0000000000000430

**Published:** 2025-11-06

**Authors:** Rui Pan, Paul L. C. Chua, Lina Madaniyazi, Chris Fook Sheng Ng, Aurelio Tobias, Qiang Guo, Vera Ling Hui Phung, Nasif Hossain, Victoria Lynch, Chau-Ren Jung, Miguel Antonio Salazar, Dominic Roye, Dung Phung, Micheline de Sousa Zanotti Stagliorio Coêlho, Paulo Hilario Nascimento Saldiva, Simon Hales, Cunrui Huang, Jan C. Semenza, Masahiro Hashizume

**Affiliations:** a Department of Global Health Policy, Graduate School of Medicine, University of Tokyo, Bunkyo-ku, Tokyo, Japan; b Department of Global Health, School of Tropical Medicine and Global Health, Nagasaki University, Nagasaki, Japan; c Institute of Environmental Assessment and Water Research, Spanish Council for Scientific Research, Barcelona, Spain; d Institute of Systems and Information Engineering, University of Tsukuba, Tsukuba, Ibaraki, Japan; e Tsukuba Institute for Advanced Research (TIAR), University of Tsukuba, Tsukuba, Ibaraki, Japan; f Department of Environmental Health Sciences, Mailman School of Public Health, Columbia University, New York City, New York; g Department of Public Health, College of Public Health, China Medical University, Taichung City, Taiwan; h Graduate School, Angeles University Foundation, Angeles, Pampanga, Philippines; i Biological Mision of Galicia (MBG) - Spanish Council for Scientific Research (CSIC), Pontevedra, Spain; j School of Public Health, University of Queensland, Brisbane, Australia; k Department of Pathology, Faculty of Medicine, University of São Paulo, São Paulo, Brazil; l Department of Public Health, University of Otago, Newtown, Wellington, New Zealand; m Vanke School of Public Health, Tsinghua University, Beijing, China; n Department of Epidemiology and Global Health , Umeå University, Umeå, Sweden; o Heidelberg Institute of Global Health, University of Heidelberg, Heidelberg, Germany

**Keywords:** Total precipitation, Diarrhea disease, Morbidity, Climate zones

## Abstract

**Background:**

Although substantial evidence has shown that precipitation is associated with diarrhea in low- and middle-income countries, few multicountry studies have focused on middle- and high-income countries to comprehensively explore the exposure–response curves between precipitation and diarrhea across different countries/regions and climate zones (tropical, arid, temperate, and cold).

**Methods:**

We collected weekly diarrhea morbidity data (primarily from hospital visits, hospital admissions, and surveillance systems) and corresponding exposure data (total precipitation and mean temperature) from 904 locations in 39 middle- and high-income countries/regions. To assess location-specific precipitation–diarrhea associations, we fitted time-stratified case-crossover analyses in distributed lag nonlinear models with 8 weeks of lag. Then, we pooled the estimates at the country/region level and by climate zone with multivariate meta-regression analysis.

**Results:**

A total of 160,266,691 diarrhea cases were included in the study. The exposure–response association curves between total precipitation and diarrhea varied substantially at the country/region level. For example, U-shaped, J-shaped, or V-shaped curves were observed in Taiwan, Hungary, Germany, Denmark, Mexico, Brazil, and Ecuador. We identified a generally increased trend of diarrhea morbidity risk in the Philippines, Czech Republic, Norway, and USA when total precipitation exceeded the minimum risk level, defined at the first percentile of total precipitation. In China, Japan, Bangladesh, and Vietnam, diarrhea morbidity risk tended to decrease with increasing total precipitation. The country-specific relative risks ranged from 1.02 (95% confidence interval [CI]: 0.88, 1.19) in Finland to 2.67 (95% CI: 2.28, 3.13) in Japan for low levels of total precipitation and from 1.02 (95% CI: 0.93, 1.11) in Ecuador to 2.03 (95% CI: 1.49, 2.76) in Austria for high levels of total precipitation. Overall, associations between precipitation and diarrhea morbidity were stronger in arid regions and weaker in cold regions.

**Conclusions:**

Associations between precipitation and diarrhea morbidity differed by country/region and climate zone. This study provides valuable evidence showing that location-specific prevention measures should be considered to mitigate the diarrhea burden associated with precipitation.

**What this study adds**

Most previous investigations quantified the association between precipitation and diarrhea in a small number of locations that covered limited geographical areas and with inconsistent results. Our finding, using global data from 904 locations in 39 middle- and high-income countries (or regions), shows substantial geographical variations with the association between weekly total precipitation and diarrhea morbidity, and the association curves were generally nonlinear. The risks of diarrheal morbidity were larger in arid regions and smaller in cold regions among the four climate zones.

## Introduction

Climate change impacts human health conditions across all stages of life, from infancy to old age, including diarrheal diseases that are climate-sensitive infectious diseases.^1,2^ In 2021, diarrheal diseases caused more than 1,238,000 deaths, particularly affecting young children and older adults, and remain the leading cause of the global burden of diseases around the world.^3,4^ With the increased incidence of extreme weather events attributed to climate change, an improved understanding of the effects of climate factors on diarrheal diseases is necessary and imperative to reduce the disease burden in the uncertain future.

Precipitation, as one of the most important climatic factors, plays a crucial role in the transmission of water-borne pathogens responsible for diarrheal diseases.^5,6^ Previous evidence has suggested that precipitation was associated with the onset and transmission of diarrheal diseases; however, the current literature has not yet provided conclusive evidence on the magnitude and direction of the association, partly owing to the underlying complex mechanism between precipitation and diarrheal diseases.^7–18^ For example, the concentration of pathogens may accumulate in the environment during dry periods and contaminate surface water following heavy rainfall or floods. Conversely, rainfall following wet periods may dilute pathogen concentrations in surface water, thereby reducing the incidence of diarrhea.^7^

Consequently, given the underlying complex mechanism of the concentration–dilution hypothesis mentioned, the association between precipitation and diarrhea could be influenced by climate zone^7^ and could differ by location, country, and region. However, most previous investigations were conducted using a small number of locations that covered limited geographical areas or had small sample sizes based on limited individual data, and the majority of the evidence came from low- and middle-income countries (LMICs) that suffer the highest burden of diarrhea mortality.^9–12,15^ The incident cases of diarrheal diseases were also prevalent in high-income countries, and the age-standardized incidence rates could increase rapidly from 2020 to 2040 in high-income countries (e.g., Japan, South Korea, and USA) partly owing to aging society due to weakened immune systems and multiple chronic diseases among older people,^19,20^ whereas there is a lack of multicountry studies to explore the precipitation–diarrhea associations including high-income countries. In addition, previous studies used different exposure definitions, study designs, and modeling approaches that could lead to heterogeneous results,^9–11,13,15,21^ and this may inhibit mechanistic understanding of the precipitation–diarrhea relationships. Therefore, an international study with highly diverse populations across multiple locations is warranted to provide comprehensive global evidence on the effects of precipitation on diarrheal diseases, which helps clarify the underlying mechanism of transmission and identify vulnerable populations with a greater need for intervention in the future.

In this study, we aimed to address the limitations of previous literature by exploring the short-term association between total precipitation and diarrheal diseases using morbidity data from 904 locations in 39 middle- and high-income countries/regions across the globe. Specifically, we examined the overall association by country/region and four types of climate zones (tropical, arid, temperate, and cold). The analysis takes advantage of a uniform analytical framework based on advanced study design and statistical methods to precisely estimate flexible exposure–response association curves and utilizes the extensive database worldwide with broad exposure contrasts to explain the spatial heterogeneity of the association.

## Methods

### Data collection

Diarrhea cases were collected from various morbidity sources, including hospital visits, hospital admissions, and surveillance systems that primarily capture medically attended cases. We included cases diagnosed with any intestinal infectious diseases (all age groups) listed in the Tenth Revision of the International Classification of Diseases: A00–A09. To maximize as many locations and cases as possible in the final analysis, we cleaned weekly time-series diarrhea morbidity data by summing all cases or all available pathogens from 904 locations in 39 countries/regions with different study periods based on the data availability. Details of time periods and data sources in each country/region are listed in Table 1 and Table S1; https://links.lww.com/EE/A383.

**Table 1.** Descriptive statistics reported by country/region and climate zone

	Locations (n)	Period	Cases	Average median of total precipitation (mm)	Average mean of temperature (ºC)
Regions					
East Asia					
China	332	2005–2020	18,200,030	11.8 (8.1)	13.5 (5.9)
Japan	47	2000–2020	20,047,736	25.4 (5.7)	13.6 (2.8)
Taiwan	20	2007–2022	4,460,359	26.1 (14.2)	21.9 (1.0)
South Asia					
Bangladesh	1	1996–2019	59,758	24.6 (NA)	25.2 (NA)
Southeast Asia					
Philippines	83	2014–2018	1,233,189	36.8 (10.9)	25.7 (1.2)
Vietnam	25	2001–2015	248,501	33.9 (8.1)	25.6 (1.9)
Central Europe					
Cyprus	1	2007–2022	2,110	1.4 (NA)	20.2 (NA)
Czech Republic	8	2018–2022	148,739	12.0 (1.2)	9.7 (0.5)
Estonia	1	2010–2022	9,149	12.7 (NA)	6.7 (NA)
Croatia	1	2019–2022	9,438	14.0 (NA)	12.8 (NA)
Hungary	20	2011–2022	139,805	9.2 (0.9)	11.9 (0.5)
Lithuania	2	2010–2019	15,818	12.8 (0.4)	7.7 (0.3)
Latvia	1	2011–2022	7,278	13.3 (NA)	7.7 (NA)
Poland	15	2017–2022	47,878	12.1 (1.1)	9.7 (0.5)
Romania	4	2011–2019	15,838	10.2 (2.3)	11.1 (1.2)
Slovenia	2	2015–2022	7,458	18.4 (1.0)	11.1 (0.2)
Slovakia	8	2010–2022	149,719	11.8 (2.5)	9.6 (1.6)
Western Europe					
Austria	9	2009–2022	98,273	19.1 (7.9)	8.5 (2.4)
Belgium	11	2011–2013	19,398	13.3 (1.1)	11.1 (0.5)
Germany	38	2012–2022	677,694	13.4 (2.2)	10.2 (0.6)
Denmark	5	2010–2019	43,811	13.0 (0.8)	9.1 (0.2)
Greece	4	2015–2022	4,776	6.4 (3.5)	16.7 (1.6)
Spain	1	2007–2022	365,462	9.2 (NA)	15.2 (NA)
Finland	4	2016–2022	3,488	11.9 (0.9)	5.5 (1.2)
France	1	2007–2022	238,456	15.0 (NA)	12.0 (NA)
Ireland	4	2011–2022	29,744	17.2 (2.3)	10.1 (0.2)
Iceland	1	2010–2022	1,468	24.1 (NA)	3.2 (NA)
Italy	4	2019–2022	20,796	11.4 (2.7)	14.4 (1.5)
Luxembourg	1	2007–2022	12,110	13.3 (NA)	9.8 (NA)
Malta	1	2007–2022	6,416	1.8 (NA)	19.4 (NA)
Netherlands	4	2014–2018	34,262	13.7 (0.3)	10.7 (0.3)
Norway	1	2007–2022	41,444	26.6 (NA)	4.8 (NA)
Portugal	5	2015–2022	9,737	4.2 (3.3)	16.2 (1.4)
Sweden	8	2016–2019	25,763	11.8 (1.4)	6.8 (2.3)
United Kingdom	9	2015–2019	345,083	13.4 (2.6)	10.3 (0.7)
North America					
USA	49	1999–2018	1,891,302	14.1 (5.5)	12.7 (4.2)
Meso-America					
Mexico	32	2000–2019	106,544,302	8.6 (6.8)	20.6 (3.6)
South America					
Brazil	117	2001–2015	4,480,260	16.8 (11.4)	23.4 (3.0)
Ecuador	24	2000–2012	569,843	49.4 (30.1)	17.7 (5.1)
Climate zones					
Tropical	204	1996–2022	37,298,859	27.9 (16.0)	25.1 (1.7)
Arid	87	1999–2022	41,044,441	3.8 (4.2)	14.0 (6.8)
Temperate	370	1999–2022	72,608,600	19.5 (11.4)	16.4 (3.7)
Cold	231	1999–2022	9,200,709	10.4 (6.0)	9.5 (3.2)
All locations	904	1996–2022	160,266,691	17.5 (13.5)	16.2 (6.8)

Average median of total precipitation, presented as mean (SD), was calculated by averaging the weekly median location-specific total precipitation across all locations during their respective study periods within each country. Average mean of temperature, presented as mean (SD), was calculated by averaging the weekly mean location-specific total precipitation across all locations during their respective study periods within each country. We excluded the polar regions (n = 12) in the main results by climate zone.

NA indicates not available.

We downloaded corresponding hourly meteorological data in each location with population density adjustment from the fifth-generation European Centre for Medium-range Weather Forecasts atmospheric reanalysis of the global climate (ERA5-Land) with a resolution of 0.1°× 0.1° (~10 × 10 km), including total precipitation and mean temperature.^22^ We first calculated daily total precipitation and mean temperature based on hourly data and then aggregated them into weekly measures for each location. To reduce the skewed distribution of total precipitation, the natural logarithm of weekly total precipitation was used in the main model to analyze the association between total precipitation and diarrhea morbidity.^15^ Weekly total precipitation values below 0.02 mm (resulting in natural logarithm values lower than −5) were set to −5 to avoid negative infinity and extreme small-value distortions.

To explain the spatial heterogeneity of the association, we further collected average levels of location-specific climatic, demographic, and socioeconomic indicators in each location. Climatic indicators of each location included the median of weekly total precipitation, weekly mean temperature calculated from ERA5-Land, and Köppen climate classification (tropical, arid, temperate, and cold regions).^23^ For locations spanning multiple climate zones, the dominant zone (with the most grids) was assigned. Other gridded demographic and socioeconomic indicators were collected from different data sources, including population density,^24^ gross domestic product (GDP),^25^ urban–rural population and land area estimates,^26^ and the global gridded relative deprivation index.^27^

### Statistical analysis

We estimated the association between weekly total precipitation and diarrhea morbidity using a two-stage design, which has been widely applied in multilocation time-series studies.^28^ In the first stage, we used a time-stratified case-crossover design to estimate the location-specific associations based on the weekly data.^29^ In the weekly time-stratified case-crossover design, we defined 4-week periods in the same year as a stratum (weeks 1–4 as first stratum, weeks 5–8 as second stratum, …, weeks 49–52/53 as 13th stratum in each year). This allows 3 or 4 control weeks for each case week in the same stratum. The study design adjusts for seasonality, long-term time trends, and unmeasured time-invariant confounders (such as sex and socioeconomic status).^30^

We used a conditional quasi-Poisson model in time-stratified case-crossover design with a distributed lag nonlinear model separately for each location:^29,31^


$$\begin{array}{l} ln\left[E\left(Y_{t.c}\right)\right]=\alpha_{c}+f\left(prec_{t,l};\beta \right)+s\left(temp;\theta \right), \end{array}$$ (Equation 1)

where E(Y) is the expected weekly diarrhea cases in week t and in time stratum c, which is every 4 weeks with same year (e.g., 1–4 weeks in 2010); α is the intercept in stratum c; f is the bidimensional spline function of exposure–lag response for the natural logarithm of total weekly precipitation and lag l; s is the natural cubic spline function of the 4-week moving average of weekly mean temperature; and β and θ are the parameters of estimation.

Specifically, we selected a quadratic B-spline function with two internal knots equally spaced at the 33rd and 66th percentiles for the location-specific logarithm of weekly total precipitation, and a natural spline function with two internal knots at equally spaced values in the log scale over 8 weeks of lag to include the delayed effects of total precipitation. The choice of knots for total precipitation was based on minimizing the quasi-Akaike information criteria in all locations (Table S2; https://links.lww.com/EE/A383), and the maximum lag weeks were guided by the observed lag-response patterns for total precipitation on diarrheal diseases that lasted for a few weeks in the previous studies and by our preliminary analysis at the first stage in each location.^32,33^ Furthermore, we included the 4-week moving average weekly mean temperature (a natural cubic spline with 3 degrees of freedom) as additional confounders in the main model.^34^ For each location, we reduced the bidimensional estimates into one dimension to obtain the cumulative exposure–response curve representing the overall association between total precipitation and diarrhea morbidity across the 8 weeks of lag.

In the second stage, we pooled the location-specific cumulative exposure–response associations using the multivariate meta-regression model into (1) country/region level, and (2) climate zone level. For the country/region level, the model was fitted with a random intercept of country/region and location (accounting for the dependence of the location-specific effects within the same country/region).^28,35^ With the pooled effect estimates, we obtained the best linear unbiased prediction of the overall cumulative exposure–response association in each country/region and location from the fitted meta-regression model.^35^ This approach can borrow information from locations with larger populations and offer more accurate estimates in locations with small diarrhea morbidity counts or short series. Then, we identified the exposure–response curve between total precipitation and diarrhea morbidity based on the best linear unbiased prediction for each country/region and location. For the climate zone level, we added climate zone as a fixed effect indicator with a random intercept of country/region and location in the meta-regression model, and then predicted the association curves by four types of climate zones (tropical, arid, temperate, and cold).

To explore local factors that might explain the variability of the associations across different locations, we added the climatic, demographic, and socioeconomic indicators as meta-predictors one at a time in the meta-regression model. We calculated the *I*^*2*^ statistics and the multivariate extension of Cochran’s *Q* statistic to measure the heterogeneity of the location-specific estimates.^36^

The risk for total precipitation on diarrhea morbidity was identified based on the predicted exposure–response association curve in each country/region, location, and climate zone. Considering the potential nonlinear exposure–response curves between total precipitation and diarrhea morbidity from previous literature and our preliminary results, we first derived the minimum risk precipitation (MRP), where the minimum percentile of diarrhea morbidity risk was identified between the 1st and 99th percentiles of the natural logarithm of weekly precipitation, from the overall cumulative nonlinear exposure–response curves. The MRP percentile was calculated as the percentile of MRP based on the distribution of total precipitation. Then we recentered the association curve at the MRP, and estimated the relative risks (RRs) and confidence intervals (CIs) for low levels of total precipitation (first percentile) and high levels of total precipitation (99th percentile) compared with the MRP.

We further estimated the attributable risk of diarrhea morbidity to total precipitation in each location using widely applied methods^37^ as:


$$\begin{array}{l} AN_{t}=N_{t}(RR_{t}-1)/RR_{t}, \end{array}$$ (Equation 2)

with *t* as the index for each week and $RR_{t}$ as the overall cumulative risk for total precipitation in week *t* compared with MRP. $N_{t}$ is the average number of diarrhea cases from 0 to 8 weeks following week *t*. $AN_{t}$ represents the weekly number of diarrhea cases attributable to total precipitation on week *t*. We obtained the total attributable number (AN) by summing the $AN_{t}$ of all locations during the entire study period, and the total attributable fraction (AF) of all locations by dividing the total AN with total diarrhea cases in each country/region. We also calculated the components attributable to low and high levels of total precipitation by summing the subsets corresponding to weeks with total precipitation lower or higher than the MRP. We did Monte Carlo simulations to derive the 95% CIs for the total AF and AN in each country/region.

To test the robustness of our findings, we conducted several sensitivity analyses. First, we increased the number of internal knots from two to three. Second, we repeated the analysis using maximum lags of 4 weeks and 12 weeks. Third, we excluded data from 2020 to 2022 to account for potential impacts of the COVID-19 pandemic.

## Results

**Figure 1.**
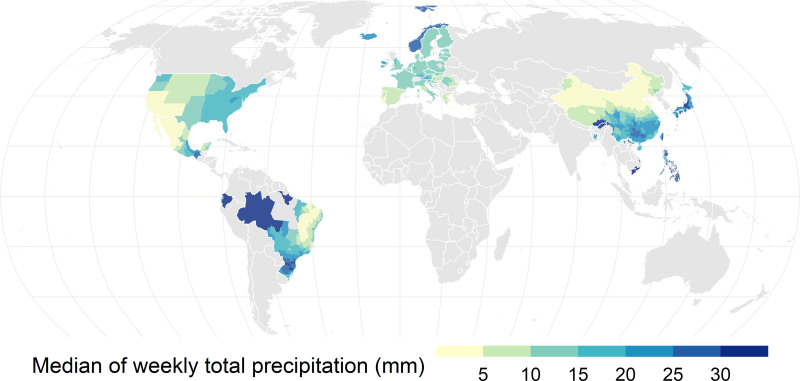
Geographical location of the 904 locations and related median of weekly total precipitation (mm) within the study period from 1996 to 2022.

The descriptive statistics of diarrhea morbidity and weather variable distribution in 39 countries/regions and four climate zones are shown in Table 1. A total of 160,266,691 diarrhea cases were included in the study. The country/region-specific weekly median of total precipitation varied accordingly from 1.4 mm in Cyprus to 49.4 mm in Ecuador, and the weekly average of the mean temperature ranged from 3.2 °C in Iceland to 25.7 °C in the Philippines. The weekly total precipitation and mean temperature were highest in tropical regions with 27.9 mm and 25.1 °C, respectively. Figure 1 displays the geographical distribution of the median of weekly total precipitation in each location. Higher levels of weekly total precipitation (over 20 mm) were observed in southern China, Japan, Taiwan, Bangladesh, the Philippines, Vietnam, the northern and southern regions of Brazil, and Ecuador.

**Figure 2.**
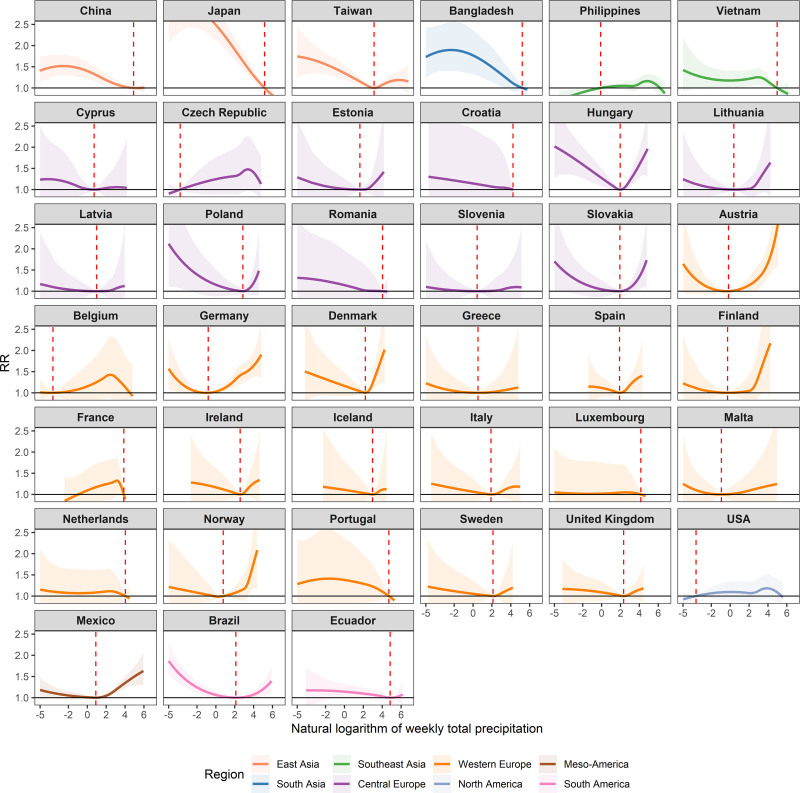
Overall cumulative exposure–response association curves, associations between the natural logarithm of weekly total precipitation and diarrhea morbidity in 39 countries/regions. Vertical lines indicate minimum risk precipitation (MRP).

The country/region-specific exposure–response association curves are shown in Figure 2. The shapes of association curves were generally nonlinear and varied considerably by country/region. For example, U-shaped, J-shaped, or V-shaped curves were observed in Taiwan, Hungary, Germany, Denmark, Mexico, Brazil, and Ecuador, indicating that both low and high levels of total precipitation increased the risk of diarrhea morbidity compared with the corresponding MRP in these countries/regions. In general, an increased risk of diarrhea morbidity with rising total precipitation was observed in the Philippines, Czech Republic, Norway, and USA when first percentile of total precipitation as MRP. Unique patterns were identified in China, Japan, Bangladesh, and Vietnam, where the risk of diarrhea morbidity decreased with higher precipitation. Large uncertainties in the association curves were noted in most European countries (e.g., Cyprus, Croatia, Latvia, Romania, Belgium, and Portugal).

**Figure 3.**
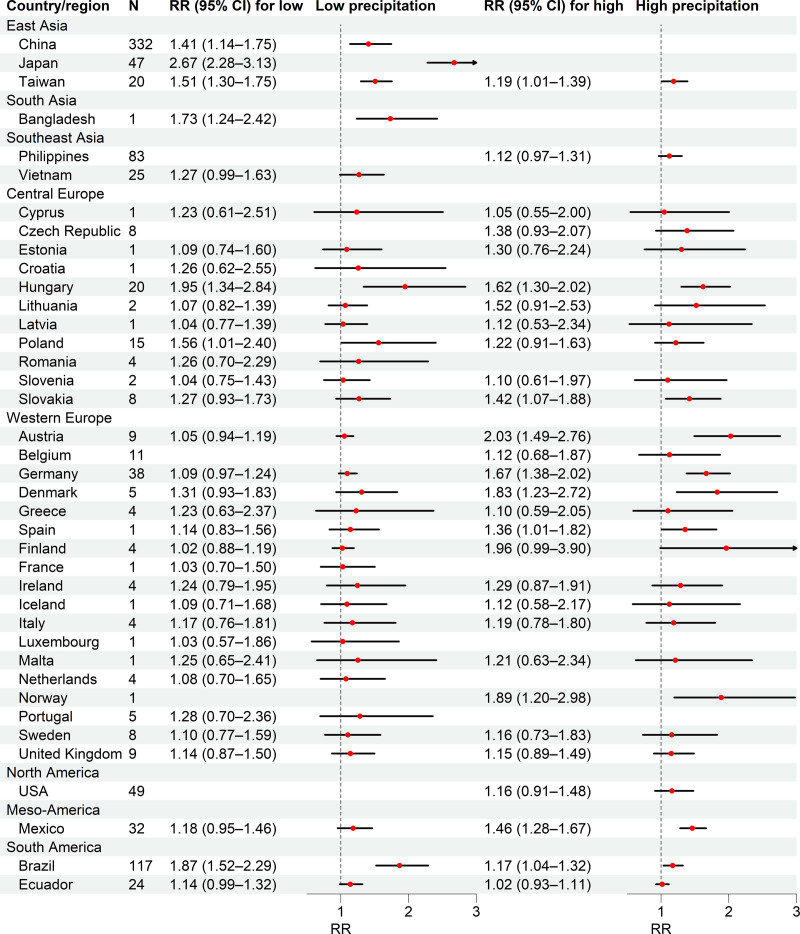
Country/region-specific and pooled relative risks (RRs, with 95% CIs) between the natural logarithm of total weekly precipitation and diarrhea morbidity for low levels of total precipitation (left, first percentile) and high levels of total precipitation (right, 99th percentile). The missing RRs in some countries, owing to the minimum risk precipitation percentiles (MRPP), were centered at the first percentile or 99th percentile.

Figure 3 and Table S3; https://links.lww.com/EE/A383 present the country/region-specific RRs for low and high levels of total precipitation. The RRs ranged from 1.02 (95% CI: 0.88, 1.19) in Finland to 2.67 (95% CI: 2.28, 3.13) in Japan for low levels of total precipitation and from 1.02 (95% CI: 0.93, 1.11) in Ecuador to 2.03 (95% CI: 1.49, 2.76) in Austria for high levels of total precipitation. Inconsistency of effect estimates was high among China (*I*^2^ = 63.80%), Japan (*I*^2^ = 54.79%), USA (*I*^2^ = 49.93%), Mexico (*I*^2^ = 63.27%), and Brazil (*I*^2^ = 63.48%), whereas heterogeneity was moderate or low among Taiwan (*I*^2^ = 20.08%), the Philippines (*I*^2^ = 27.63%), Vietnam (*I*^2^ = 39.17%), and most European countries (e.g., Hungary, Austria, Belgium, Germany, and the United Kingdom). The location-specific RRs are shown in Figure S1; https://links.lww.com/EE/A383, and we observed larger RRs for low levels of total precipitation in southwest and central China, Japan, Taiwan, and southern Brazil, and higher RRs for high levels of total precipitation in some European countries, Mexico, and northeast Brazil.

Table S4; https://links.lww.com/EE/A383 shows the estimated AF calculated as total and as separated components caused by low and high levels of total precipitation in each country/region. Overall, the total AF of morbidity varied substantially among countries/regions, and a notably high total AF with 39.55% (95% CI: 11.16%, 57.75%) was observed in Japan. The total AF as separated components caused by low and high levels of total precipitation also differed by country/region, and the CIs for most countries/regions were wide, partly owing to the high heterogeneity of the association curves for each location within each country/region.

**Figure 4.**
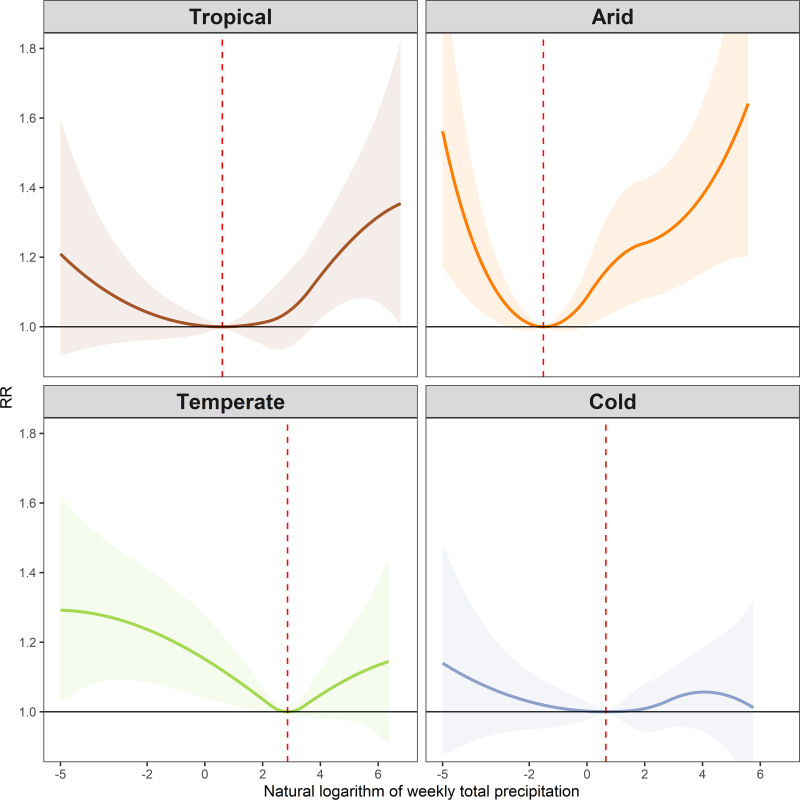
Overall cumulative exposure–response association curves between the natural logarithm of weekly total precipitation and diarrhea morbidity in tropical (n = 204), arid (n = 87), temperate (n = 370), and cold regions (n = 231). Vertical lines indicate minimum risk precipitation (MRP).

The association curves predicted by four climate zone types are shown in Figure 4 and Table S5; https://links.lww.com/EE/A383. In general, the MRP was relatively higher in temperate regions (48th percentile, 17.5 mm) than in tropical (16th percentile, 1.8 mm), arid (16th percentile, 0.2 mm), and cold regions (21st percentile, 1.9 mm). Compared with corresponding MRPs, the relatively higher RRs for low levels of total precipitation with RR of 1.56 (95% CI: 1.17, 2.08) and for high levels of total precipitation with RR of 1.41 (95% CI: 1.17, 1.71) were observed in arid regions. We found significant risks for high levels of total precipitation with RR of 1.26 (95% CI: 1.08, 1.46) in tropical regions and for low levels of total precipitation with RR of 1.29 (95% CI: 1.05, 1.59) in temperate regions. The RRs were relatively lower and not statistically significant in cold regions and were estimated to be 1.14 (95% CI: 0.88, 1.48) for low levels of total precipitation and 1.05 (95% CI: 0.92, 1.21) for high levels of total precipitation, respectively. High spatial heterogeneity (*I*^2^ > 50%) was observed across the four different climate zone types.

In the multivariate meta-regression model, we observed substantial heterogeneity (*I*^2^ = 65.25%) in the total precipitation–diarrhea association among 904 locations with a significant multivariate Cochran’s Q test (*P <* 0.001) (Table S6; https://links.lww.com/EE/A383). The residual heterogeneity reduced to 61.15% in the one predictor meta-regression model with climate zone as fixed effects, and the Wald test suggested that the association between total precipitation and diarrhea varied across climate zones (*P <* 0.001). Although the Wald test results showed that the mean of weekly temperature, population density, and GDP would play significant roles in the modification of the total precipitation–diarrhea association (*P <* 0.001), the residual heterogeneity reduced a little compared with the model with intercept only.

In the sensitivity analysis, the results remained generally robust after changing the number of internal knots from two to three and excluding the COVID-19 outbreak period. The estimates were generally lower across most countries/regions when we used 4 weeks as the maximum lag, and the estimates were slightly larger in some countries (e.g., China, Japan, Germany, Brazil, and Ecuador) accompanied by wider CIs when the maximum lag was 12 weeks (Table S7; https://links.lww.com/EE/A383).

## Discussion

To the best of our knowledge, this is the most extensive study to comprehensively investigate the exposure–response association curves of the effects of total precipitation on diarrhea morbidity across 904 locations in 39 middle- and high-income countries/regions with diverse climate zones and a substantially large sample size. Our findings indicated that precipitation was associated with diarrhea morbidity, including in some high-income countries/regions. The association curves between weekly total precipitation and diarrhea morbidity were generally nonlinear and could be clustered from four main aspects when compared with corresponding MRP: (1) the U-shape, J-shape, and V-shape, (2) a generally increasing trend, (3) a generally decreasing trend, and (4) an unclear shape or shape with large uncertainty. The effects of total precipitation on diarrhea morbidity varied across climate zones, with larger effects observed in arid regions and smaller effects in cold regions.

This study provides an essential contribution to the literature on the diarrhea morbidity risks associated with short-term total precipitation exposure. The association curves observed in the present study suggest that both low and high levels of total precipitation are linked to increased diarrhea morbidity in middle- and high-income countries/regions. Notably, these associations varied by country/region, partially aligning with findings from previous studies. Sufficient previous studies have explored the risk of diarrheal diseases with precipitation, primarily in LMICs, and a positive association between precipitation or rainfall and diarrheal diseases was reported in Bangladesh,^38,39^ Nepal,^14^ Brazil,^40^ India,^41^ Cambodia,^42^ Vietnam,^43^ USA,^44^ South Korea,^17^ Indonesia,^9^ China,^45^ 18 Pacific Island countries,^46^ New Zealand,^47^ and some African countries (e.g., Mozambique, Kenya, and Senegal).^18,48,49^ Two recent studies based on Demographic and Health Survey data also reported that high total precipitation anomalies were associated with an increased risk of children’s diarrhea in over 30 LMICs.^12,33^ The nonlinear, J- or U-shaped association curves between total precipitation/rainfall and all or pathogen-specific diarrheal diseases were observed by Epstein et al.,^21^ and Fang et al.^45^ Ikeda et al.,^50^ and Perez-Corrales et al.^51^ found a reduction in the risk of diarrhea with increased total precipitation in Rural Limpopo and Costa Rican children, respectively. Several studies have shown no significant association between total precipitation and diarrhea.^52–54^ When comparing the present study with previous research, caution is needed because of differences in study design (e.g., cross-section, Bayesian spatial study, and time-series study), exposure definition (e.g., total precipitation vs. rainfall, or daily, weekly, monthly total or average exposure, and extreme exposure), health outcome (e.g., all-cause vs. pathogen-specific diarrhea, mortality vs. morbidity data), age group (e.g., all age group vs. children), and sample size (e.g., individual-level vs. time-series data).

One notable finding of this study is the exploration of association curve shapes across four climate zone types, where we observed a larger effect of total precipitation on diarrhea morbidity in arid regions and a smaller effect in cold regions. We should interpret this result carefully considering the high heterogeneity in each climate zone. For example, the RRs in cold regions were relatively lower and not statistically significant in our pooled estimates, whereas some studies in cold climate zones have reported significant associations between precipitation and diarrheal disease,^55,56^ indicating that precipitation-related diarrheal risk in cold climates may be context-dependent and influenced by local environmental and infrastructural factors. We hypothesize that the higher risk associated with total precipitation in arid regions could be explained by a lack of clean water and the concentration of enteric pathogens during dry spells. In contrast, the smaller effect in cold areas could be attributed to precipitation more commonly occurring as snow rather than rain, where pathogen flushing may be influenced by seasonal shifts in temperature.^57^ Limited previous studies have explored whether the effects of precipitation on diarrheal diseases differed in different climate zones. Yang et al.^11^ showed that extreme rainfall had a negative association with the incidence rate of dysentery in alpine plateau regions, but not in temperate, subtropical monsoon, and tropical monsoon regions in China. Dimitrova et al.^33^ also found that droughts increased the risk of diarrhea in the tropical savanna regions, whereas heavy total precipitation events were positively associated with diarrhea in humid subtropical regions. Our findings are partially consistent with a recent study that reported an increased risk of diarrhea mortality associated with higher precipitation in tropical regions and a U-shaped relationship in temperate and arid regions.^58^ To clarify whether climate zones would modify the effects of total precipitation on diarrheal diseases, more studies are warranted to further explore this issue.

The mechanism behind the association between precipitation and diarrheal diseases has not yet been clearly established. Carlton et al.^8^ and Levy et al.^5^ suggested a “concentration-dilution hypothesis,” and Kraay et al.^7^ extended this hypothesis more specifically as follows: pathogens would concentrate in the environment during dry periods with low levels of precipitation, then extreme rain or flooding increases the diarrhea incidence by flushing pathogens into surface water, overwhelming sanitation, and damaging the infrastructure; in contrast, precipitation following a wet period may lead lower diarrhea risk by diluting pathogen concentrations. The main findings observed in the present study could well be explained by the aforementioned mechanism. The relatively higher and significant RRs for high levels of total precipitation in tropical and arid areas could be explained by the high prevalence of extreme rainfall events in tropical regions following the dry season and the concentration of pathogens in a dry environment in arid regions. Additionally, the risk of diarrhea may be exacerbated by a lack of clean water during drought events in arid regions. We assumed that the relatively higher MRP in temperate areas could partly be explained by a regular flush in wet periods that would cause a lower risk. The complex mechanisms mentioned earlier suggest that many other factors (e.g., weather factors, climate zones and GDP in this study, or types of pathogens, dietary habits, and behavior culture that are not included in the present study) can modify the associations and partly explain the different exposure–response association curves and high heterogeneity of results across locations and countries/regions in the present study. For example, lower GDP areas may lack infrastructure such as sewage systems and water treatment, leading to higher exposure to contaminated water during heavy rain or drought.

There are several advantages to this global study. First, this study used a large surveillance and hospitalization dataset covering 904 locations in 39 countries/regions with a large sample size, providing significant statistical power and stability for the findings, while also offering new and robust evidence from multiple high-income countries. Second, the advanced and uniform analytical approaches can aid in integrating and comparing the results across different locations, countries, regions, and populations. Third, a global study with diverse climate zones helps us to identify the potential mechanism underlying the associations. Fourth, morbidity data captures some mild and moderate cases, and not just the most severe outcomes, compared with mortality data, which aligns better with public health goals of prevention and improving quality of life rather than solely reducing deaths. In summary, the present study has well addressed some limitations of previous studies as reviewed by Kraay et al.^7^

Several limitations need to be acknowledged. One of the most notable limitations of our study is the heterogeneity in the total diarrhea morbidity data source across the 39 countries/regions. This variability likely stems from differences in surveillance systems, diagnostic capacities, and case definitions, which may have led to potential inconsistencies in outcome classification. Our dataset primarily comprises medically attended diarrhea cases, which would also may underrepresent mild cases that do not lead to healthcare visits, and the proportion of mild cases captured likely varies by region due to differences in healthcare accessibility, health-seeking behavior, and cultural norms. We acknowledge this variability as one important limitation of this cross-country epidemiological research relying on morbidity data. Nonetheless, in this global-scale analysis, the country/region-specific morbidity data represent the best available and most comprehensive indicators of total diarrheal disease burden within each respective country/region. Despite differences in diagnostic criteria and procedures, these data reliably reflect broader trends in total diarrhea morbidity and are routinely used in public health monitoring and international comparative studies. Additionally, it is reasonable to expect that the distribution of diarrheal pathogens varies by country/region due to climatic, environmental, infrastructural differences and other contextual factors. Therefore, while outcome heterogeneity may introduce some variability in the effect estimates, we suggest that it is unlikely to substantially alter the validity of our broader findings in addressing the study objectives. However, aggregating all diarrheal pathogens into a single “all-cause diarrhea” category may obscure pathogen-specific differences in the precipitation–diarrhea relationship. This is particularly relevant given variations in incubation periods, environmental persistence, and transmission pathways among bacterial, viral, and protozoan pathogens. We also suggested that future research using standardized case definitions or harmonized pathogen-specific data would be valuable to build upon our findings and better elucidate the underlying mechanisms. Second, a time-series design based on ecological data with aggregated exposure would lead to Berkson’s bias with the correct point estimates and an inflation of the standard errors.^59^ Third, although this study provided risk summaries across four inhabited continents, our results cannot truly represent global estimates, given that some areas, especially in low-income countries, were underrepresented or not assessed. Fourth, the total precipitation based on ERA5-land is modeled through parameterizations, which means that the total precipitation exposure may differ from observed values, especially in the case of warm-season extremes. Fifth, our study does not explicitly distinguish between antecedent rainfall conditions (e.g., rainfall following dry vs. wet periods), which may play a critical role in pathogen mobilization and exposure risk. Future studies could improve upon this by incorporating indicators of prior rainfall accumulation, rainfall anomalies, or drought indices to better capture antecedent hydrological conditions relevant to the concentration–dilution hypothesis. Finally, the study was conducted using aggregated time-series data, which makes it hard to provide a more refined analysis of potential biological mechanisms and differential susceptibility patterns at the individual level.

In summary, our findings indicated that both low and high levels of total precipitation increased the risk of diarrhea morbidity, with substantial variations across locations and countries/regions. The evidence in our study provides important policy that healthcare providers should be aware of the increased risk of diarrhea cases not only during heavy rainfall events but also during drought periods, and corresponding adaptation measures during different extreme weather events in response to reduce the attributable diarrhea cases from precipitation are crucial in the context of climate change.^1^ Policymakers should improve decision-making for diarrhea prevention programs that location-specific targeted measurements and interventions should be taken for different vulnerable populations in different countries/regions, including in high-income countries/regions. Attention should be given to future researchers that the climate zone was an important indicator and should be considered in future projections of the diarrhea burden under various climate change scenarios. More research is warranted to better understand the complex causality or effect modification between precipitation and diarrhea (e.g., to explore the risk of pathogen-specific diarrheal diseases, and the different infection pathways via livestock, water, and food; and effect modification by season, prior precipitation level, and disease susceptibility factors). This study has important implications for diarrhea prevention programs to minimize diarrhea cases due to total precipitation and help in future projections of diarrhea burden under climate change.

## Conflicts of interest statement

The authors declare that they have no conflicts of interest with regard to the content of this report.

## Supplementary Material
