# Supplementary material for: The association between total precipitation and diarrhea morbidity: A multicountry study across diverse climate zones
Source: Environ Epidemiol. 2025 Nov 6;9(6):e430. doi: 10.1097/EE9.0000000000000430 (PMC12594309; doi:10.1097/EE9.0000000000000430)
Supplement: Supplementary file 1 [file ee9-9-e430-s001.pdf]

Supplemental Materials

The Association between Total Precipitation and Diarrhea Morbidity: A Multi-Country Study across Diverse Climate Zones

Lists of the supplementary table and figures

Table S1. Details of data sources in 39 countries/regions 2

Table S2. The sum of Q-AIC over 904 locations in different models 5

Table S3. Country/region-specific RRs (95% CIs) for the low and high levels of total precipitation..........6

Table S4. Country/region-specific attributable fraction (95% CIs) for the low and high levels of total precipitation 8

Table S5. Climate zone-specific RRs (95% CIs) for the low and high levels of total precipitation...........10

Table S6. Meta-regression models for explaining variation in overall precipitation effects: Wald test, Cochran Q test for heterogeneity, I^2 statistics for residual heterogeneity...11

Table S7. Country/region-specific RRs (95% CIs) for the low level and high levels of total precipitation in different sensitivity analysis 12

Figure S1. The location-specific RRs for low and high levels of total precipitation shown as spatial map. *RR, relative risk.* 14

Table S1. Details of data sources in 39 countries/regions

Country	Source type	Database / Agency	Data resolution	Pathogens/ICD codes
China	diarrhea surveillance	Chinese Center for Disease Control and Prevention	City level	*Shigella*, typhoid, cholera, other infectious
Japan	infectious gastroenteritis surveillance	National Institute of Infectious Diseases	Prefecture level	Infectious gastroenteritis
Taiwan	diarrhea surveillance	Taiwan Centers for Disease Control	City level	Acute diarrhea
Bangladesh	diarrhea hospital visits	International Centre for Diarrheal Disease Research, Bangladesh	Division level	Diarrheal diseases
Philippines	health insurance for hospital admissions	Philippine Health Insurance Corporation	Province level	ICD 10: A00–A09
Vietnam	diarrhea hospital admissions	Individual health facilities	Province level	ICD 10: A00–A09
Cyprus	diarrhea surveillance	The European Surveillance System	NUTS level 0	*Campylobacter*, *Cryptosporidium*, giardia, salmonella, enterotoxigenic *E coli*, yersinia
Czech Republic	diarrhea surveillance	The European Surveillance System	NUTS level 2	*Campylobacter*, *Cryptosporidium*, giardia, salmonella, enterotoxigenic *E coli*, yersinia
Estonia	diarrhea surveillance	The European Surveillance System	NUTS level 0	*Campylobacter*, *Cryptosporidium*, giardia, salmonella, enterotoxigenic *E coli*, yersinia
Croatia	diarrhea surveillance	The European Surveillance System	NUTS level 0	*Campylobacter*, *Cryptosporidium*, giardia, salmonella, enterotoxigenic *E coli*, yersinia
Hungary	diarrhea surveillance	The European Surveillance System	NUTS level 3	*Campylobacter*, *Cryptosporidium*, giardia, salmonella, enterotoxigenic *E coli*, yersinia
Lithuania	diarrhea surveillance	The European Surveillance System	NUTS level 2	*Campylobacter*, *Cryptosporidium*, giardia, salmonella, enterotoxigenic *E coli*, yersinia
Latvia	diarrhea surveillance	The European Surveillance System	NUTS level 0	*Campylobacter*, *Cryptosporidium*, giardia, salmonella, enterotoxigenic *E coli*, yersinia
Poland	diarrhea surveillance	The European Surveillance System	NUTS level 2	*Campylobacter*, *Cryptosporidium*, giardia, salmonella, enterotoxigenic *E coli*, yersinia
Romania	diarrhea surveillance	The European Surveillance System	NUTS level 1	*Campylobacter*, *Cryptosporidium*, giardia, salmonella, enterotoxigenic *E coli*, yersinia
Slovenia	diarrhea surveillance	The European Surveillance System	NUTS level 2	*Campylobacter*, *Cryptosporidium*, giardia, salmonella, enterotoxigenic *E coli*, yersinia
Slovakia	diarrhea surveillance	The European Surveillance System	NUTS level 3	*Campylobacter*, *Cryptosporidium*, giardia, salmonella, enterotoxigenic *E coli*, yersinia
Austria	diarrhea surveillance	The European Surveillance System	NUTS level 2	*Campylobacter*, *Cryptosporidium*, giardia, salmonella, enterotoxigenic *E coli*, yersinia

Belgium	diarrhea surveillance	The European Surveillance System	NUTS level 2	*Campylobacter*, *Cryptosporidium*, giardia, salmonella, enterotoxigenic *E coli*, yersinia
Germany	diarrhea surveillance	The European Surveillance System	NUTS level 2	*Campylobacter*, *Cryptosporidium*, giardia, salmonella, enterotoxigenic *E coli*, yersinia
Denmark	diarrhea surveillance	The European Surveillance System	NUTS level 2	*Campylobacter*, *Cryptosporidium*, giardia, salmonella, enterotoxigenic *E coli*, yersinia
Greece	diarrhea surveillance	The European Surveillance System	NUTS level 1	*Campylobacter*, *Cryptosporidium*, giardia, salmonella, enterotoxigenic *E coli*, yersinia
Spain	diarrhea surveillance	The European Surveillance System	NUTS level 0	*Campylobacter*, *Cryptosporidium*, giardia, salmonella, enterotoxigenic *E coli*, yersinia
Finland	diarrhea surveillance	The European Surveillance System	NUTS level 2	*Campylobacter*, *Cryptosporidium*, giardia, salmonella, enterotoxigenic *E coli*, yersinia
France	diarrhea surveillance	The European Surveillance System	NUTS level 0	*Campylobacter*, *Cryptosporidium*, giardia, salmonella, enterotoxigenic *E coli*, yersinia
Ireland	diarrhea surveillance	The European Surveillance System	NUTS level 3	*Campylobacter*, *Cryptosporidium*, giardia, salmonella, enterotoxigenic *E coli*, yersinia
Iceland	diarrhea surveillance	The European Surveillance System	NUTS level 0	*Campylobacter*, *Cryptosporidium*, giardia, salmonella, enterotoxigenic *E coli*, yersinia
Italy	diarrhea surveillance	The European Surveillance System	NUTS level 1	*Campylobacter*, *Cryptosporidium*, giardia, salmonella, enterotoxigenic *E coli*, yersinia
Luxembourg	diarrhea surveillance	The European Surveillance System	NUTS level 0	*Campylobacter*, *Cryptosporidium*, giardia, salmonella, enterotoxigenic *E coli*, yersinia
Malta	diarrhea surveillance	The European Surveillance System	NUTS level 0	*Campylobacter*, *Cryptosporidium*, giardia, salmonella, enterotoxigenic *E coli*, yersinia
Netherlands	diarrhea surveillance	The European Surveillance System	NUTS level 1	*Campylobacter*, *Cryptosporidium*, giardia, salmonella, enterotoxigenic *E coli*, yersinia
Norway	diarrhea surveillance	The European Surveillance System	NUTS level 0	*Campylobacter*, *Cryptosporidium*, giardia, salmonella, enterotoxigenic *E coli*, yersinia
Portugal	diarrhea surveillance	The European Surveillance System	NUTS level 2	*Campylobacter*, *Cryptosporidium*, giardia, salmonella, enterotoxigenic *E coli*, yersinia
Sweden	diarrhea surveillance	The European Surveillance System	NUTS level 2	*Campylobacter*, *Cryptosporidium*, giardia, salmonella, enterotoxigenic *E coli*, yersinia
United Kingdom	diarrhea surveillance	The European Surveillance System	NUTS level 1	*Campylobacter*, *Cryptosporidium*, giardia, salmonella, enterotoxigenic *E coli*, yersinia
USA	diarrhea surveillance	Centers for Disease Control and Prevention	State level	*Cryptosporidium*, enterotoxigenic *E. coli*, giardia, salmonella, *Shigella*
Mexico	diarrhea surveillance	Dirección General de Epidemiología	State level	ICD 10: A00–A09

Brazil	diarrhea notifications	Instituto Brasileiro de Geografia e Estatística	Intermediate level	ICD 10: A00–A09
Ecuador	diarrhea hospital admissions	Instituto Nacional de Estadística y Censos	Province level	ICD 10: A00–A09

Note: NUTS, Nomenclature of territorial units for statistics.

Table S2. The sum of Q-AIC over 904 locations in different models

**Model**	**Knots of location**	**Temperature adjustment**	**Sum of *Q*-AIC**
Main model	33rd, 66th	Running mean of 4 weeks	7 272 543
Model 1	25th, 50th, 75th	Running mean of 4 weeks	7 276 314
Model 2	10th, 75th, 90th	Running mean of 4 weeks	7 283 209
Model 3	33rd, 66th	Current week temperature	7 379 521

Note: Q-AIC, quasi-Akaike information criteria.

Table S3. Country/region-specific RRs (95% CIs) for the low and high levels of total precipitation

Country/region	Locations (n)	MRPP	MRP (mm)	RR for low precipitation	RR for high precipitation	I^2 (%)	*P*–value
China	332	99	134·92	1·41 (1·14-1·75)	NA	63·80	<0·001
Japan	47	99	174·5	2·67 (2·28-3·13)	NA	54·79	<0·001
Taiwan	20	48	23·05	1·51 (1·30-1·75)	1·19 (1·01-1·39)	20·08	0·07
Bangladesh	1	99	183.99	1.73 (1.24-2.42)	NA	NA	NA
Philippines	83	1	0.9	NA	1.12 (0.97-1.31)	27.63	<0.001
Vietnam	25	99	144.57	1.27 (0.99-1.63)	NA	39.17	<0.001
Cyprus	1	55	2.07	1.23 (0.61-2.51)	1.05 (0.55-2.00)	NA	NA
Czech Republic	8	1	0.02	NA	1.38 (0.93-2.07)	0.00	0.84
Estonia	1	22	5.13	1.09 (0.74-1.60)	1.30 (0.76-2.24)	NA	NA
Croatia	1	99	68.98	1.26 (0.62-2.55)	NA	NA	NA
Hungary	20	42	7.13	1.95 (1.34-2.84)	1.62 (1.30-2.02)	29.37	0.01
Lithuania	2	7	1.47	1.07 (0.82-1.39)	1.52 (0.91-2.53)	0.00	0.41
Latvia	1	13	2.69	1.04 (0.77-1.39)	1.12 (0.53-2.34)	NA	NA
Poland	15	69	17.48	1.56 (1.01-2.40)	1.22 (0.91-1.63)	18.61	0.12
Romania	4	99	56.78	1.26 (0.70-2.29)	NA	33.06	0.12
Slovenia	2	11	1.54	1.04 (0.75-1.43)	1.10 (0.61-1.97)	0.00	0.43
Slovakia	8	33	6.91	1.27 (0.93-1.73)	1.42 (1.07-1.88)	5.84	0.38
Austria	9	4	0.83	1.05 (0.94-1.19)	2.03 (1.49-2.76)	20.73	0.15
Belgium	11	1	0.03	NA	1.12 (0.68-1.87)	32.45	0.03
Germany	38	3	0.44	1.09 (0.97-1.24)	1.67 (1.38-2.02)	14.23	0.08
Denmark	5	37	9.12	1.31 (0.93-1.83)	1.83 (1.23-2.72)	13.01	0.3
Greece	4	28	1.69	1.23 (0.63-2.37)	1.10 (0.59-2.05)	48.46	0.03
Spain	1	40	6.67	1.14 (0.83-1.56)	1.36 (1.01-1.82)	NA	NA
Finland	4	4	0.76	1.02 (0.88-1.19)	1.96 (0.99-3.90)	17.23	0.27
France	1	99	47.77	1.03 (0.70-1.50)	NA	NA	NA
Ireland	4	40	13.11	1.24 (0.79-1.95)	1.29 (0.87-1.91)	0.00	0.88
Iceland	1	39	19.58	1.09 (0.71-1.68)	1.12 (0.58-2.17)	NA	NA
Italy	4	38	6.71	1.17 (0.76-1.81)	1.19 (0.78-1.80)	30.87	0.14
Luxembourg	1	99	63.73	1.03 (0.57-1.86)	NA	NA	NA

Malta	1	35	0.39	1.25 (0.65-2.41)	1.21 (0.63-2.34)	NA	NA
Netherlands	4	99	56.36	1.08 (0.70-1.65)	NA	15.76	0.29
Norway	1	1	2.18	NA	1.89 (1.20-2.98)	NA	NA
Portugal	5	99	110.52	1.28 (0.70-2.36)	NA	33.43	0.09
Sweden	8	37	8.29	1.10 (0.77-1.59)	1.16 (0.73-1.83)	0.00	0.58
United Kingdom	9	40	10.31	1.14 (0.87-1.50)	1.15 (0.89-1.49)	0.00	0.93
USA	49	1	0.03	NA	1.16 (0.91-1.48)	49.93	<0.001
Mexico	32	34	2.47	1.18 (0.95-1.46)	1.46 (1.28-1.67)	63.27	<0.001
Brazil	117	39	8.29	1.87 (1.52-2.29)	1.17 (1.04-1.32)	63.48	<0.001
Ecuador	24	94	126.61	1.14 (0.99-1.32)	1.02 (0.93-1.11)	36.01	<0.001

Note: RR, relative risk; CIs, confidence intervals; MRPP, minimum risk precipitation percentile; MRP, minimum risk precipitation; NA, not available. The NA value of RRs in some countries owing the minimum risk precipitation percentiles (MRPP) were centered at 1st percentile or 99th percentile.

Table S4. Country/region-specific attributable fraction (95% CIs) for the low and high levels of total precipitation

Country/region	AF for total (%)	AF for low levels (%)	AF for low levels (%)
China	23.04 (-8.77, 42.64)	15.52 (-3.03, 27.49)	7.52 (-6.59, 15.75)
Japan	39.55 (11.16, 57.75)	39.52 (11.16, 57.71)	0.02 (-0.17, 0.20)
Taiwan	13.22 (-6.60, 26.73)	9.75 (-6.17, 20.79)	3.48 (-2.09, 7.80)
Bangladesh	22.23 (13.32, 29.08)	21.68 (12.67, 28.58)	0.56 (-0.73, 1.74)
Philippines	23.79 (-23.01, 50.34)	7.30 (-11.28, 17.00)	16.48 (-11.72, 33.53)
Vietnam	22.29 (-22.95, 46.74)	19.70 (-17.73, 40.32)	2.59 (-5.33, 7.57)
Cyprus	18.14 (-16.45, 35.78)	15.02 (-11.84, 29.93)	3.12 (-12.43, 12.05)
Czech Republic	31.51 (-46.03, 65.90)	5.67 (-8.73, 12.26)	25.84 (-36.59, 53.30)
Estonia	8.21 (-22.86, 27.81)	0.03 (-1.15, 0.96)	8.18 (-22.58, 29.10)
Croatia	18.34 (-96.38, 61.64)	18.34 (-102.09, 61.29)	NA
Hungary	15.30 (-1.33, 27.34)	5.51 (-2.61, 11.39)	9.79 (-3.02, 18.86)
Lithuania	15.00 (-18.80, 36.06)	0.32 (-3.30, 3.07)	14.68 (-21.01, 35.10)
Latvia	10.40 (-46.56, 41.56)	0.01 (-0.37, 0.34)	10.39 (-51.14, 40.73)
Poland	5.85 (-15.04, 19.45)	3.87 (-14.88, 16.60)	1.97 (-7.41, 8.28)
Romania	15.94 (-54.83, 46.93)	14.70 (-52.16, 43.52)	1.23 (-5.84, 5.49)
Slovenia	11.78 (-69.07, 51.60)	4.83 (-36.23, 23.97)	6.95 (-34.91, 29.30)
Slovakia	8.48 (-10.32, 22.03)	1.47 (-6.54, 7.25)	7.01 (-6.79, 17.35)
Austria	23.22 (-5.21, 42.84)	0.08 (-0.46, 0.53)	23.14 (-5.55, 42.69)
Belgium	27.61 (-55.87, 62.61)	10.53 (-12.15, 21.00)	17.08 (-37.44, 42.36)
Germany	25.71 (-3.99, 45.37)	0.04 (-0.46, 0.42)	25.67 (-3.72, 44.98)
Denmark	17.58 (-2.71, 32.04)	3.27 (-2.65, 7.36)	14.31 (-3.44, 26.58)
Greece	11.05 (-29.99, 33.65)	5.35 (-17.92, 18.35)	5.69 (-21.13, 20.20)
Spain	8.93 (-1.18, 17.16)	2.41 (-3.23, 7.57)	6.53 (0.52, 11.38)
Finland	18.14 (-30.88, 44.42)	0.06 (-1.01, 0.84)	18.08 (-29.74, 46.43)
France	34.09 (-1.00, 57.21)	34.09 (0.88, 56.01)	NA
Ireland	11.23 (-9.67, 25.59)	4.33 (-6.62, 11.74)	6.90 (-8.23, 17.51)
Iceland	3.84 (-28.29, 22.17)	1.49 (-12.69, 11.38)	2.35 (-23.76, 16.72)
Italy	12.91 (-41.86, 40.14)	8.74 (-45.01, 32.31)	4.17 (-3.16, 9.11)
Luxembourg	9.46 (-82.67, 52.38)	NA	9.46 (-83.97, 56.00)
Malta	11.06 (-39.62, 37.37)	6.88 (-21.57, 17.11)	4.18 (-33.84, 26.26)
Netherlands	15.94 (-35.83, 46.99)	9.50 (-27.02, 29.95)	6.44 (-9.19, 15.54)

Norway	32.41 (5.90, 50.02)	NA	32.41 (4.99, 50.52)
Portugal	31.67 (-69.97, 69.19)	19.14 (-21.57, 34.99)	12.53 (-44.08, 35.07)
Sweden	6.72 (-42.96, 31.70)	0.82 (-8.21, 7.05)	5.90 (-40.76, 28.33)
United Kingdom	8.56 (-21.84, 28.00)	4.41 (-14.69, 15.39)	4.15 (-10.33, 14.05)
USA	20.72 (-23.86, 46.65)	8.00 (-14.78, 20.94)	12.73 (-10.07, 26.38)
Mexico	15.14 (-0.27, 26.81)	1.80 (-1.12, 4.14)	13.34 (-0.76, 23.74)
Brazil	14.72 (-4.77, 28.95)	7.25 (-4.69, 15.40)	7.47 (-2.45, 15.12)
Ecuador	14.97 (-17.20, 35.19)	8.88 (-10.31, 20.96)	6.09 (-7.11, 15.40)

Note: CIs, confidence intervals; AF, attributable fraction; NA, not available.

Table S5. Climate zone-specific RRs (95% CIs) for the low and high levels of total precipitation

Climate zone	Locations (n)	MRPP	MRP (mm)	RR for low precipitation	RR for high precipitation	I^2 (%)	*P*–value
Tropical	204	16	1.8	1.21 (0.92-1.60)	1.26 (1.08-1.46)	58.34	<0.001
Arid	87	16	0.2	1.56 (1.17-2.08)	1.41 (1.17-1.71)	66.57	<0.001
Temperate	370	48	17.5	1.29 (1.05-1.59)	1.10 (0.97-1.24)	63.95	<0.001
Cold	231	21	1.9	1.14 (0.88-1.48)	1.05 (0.92-1.21)	56.56	<0.001

Note: RR, relative risk; CIs, confidence intervals; MRPP, minimum risk precipitation percentile; MRP, minimum risk precipitation.

Table S6. Meta-regression models for explaining variation in overall precipitation effects: Wald test, Cochran Q test for heterogeneity, I^2 statistics for residual heterogeneity

Predictor	Test for predictor[a]	*Q*-test[b]	*I2 (%)*
Intercept only		<0.001	65.25
avgtmean	<0.001	<0.001	63.80
midprec	0.26	<0.001	64.76
clim	<0.001	<0.001	61.15
pop	<0.001	<0.001	64.89
gdp	<0.001	<0.001	65.20
urban	0.12	<0.001	65.19
grdi	0.20	<0.001	65.24

[a] P-value for the multivariate Wald test for the significance of the meta predictors.

[b] P-value for the Cochran Q-test.

Note: avgtmean, weekly mean temperature; midprec, median of weekly total precipitation; clim, climate zone; pop, population density; gdp, gross domestic product; urban, urban-rural population and land area estimates; grdi, the global gridded relative deprivation index.

Table S7. Country/region-specific RRs (95% CIs) for the low level and high levels of total precipitation in different sensitivity analysis

Country/region	Main model		3 Knots		lag04		lag12		COVID	
	RR for low precipitation	RR for high precipitation	RR for low precipitation	RR for high precipitation	RR for low precipitation	RR for high precipitation	RR for low precipitation	RR for high precipitation	RR for low precipitation	RR for high precipitation
China	1.41 (1.14-1.75)	NA	1.42 (1.13-1.78)	NA	1.46 (1.27-1.67)	NA	1.50 (1.11-2.03)	NA	1.15 (0.88-1.50)	NA
Japan	2.67 (2.28-3.13)	NA	2.72 (2.31-3.20)	NA	1.36 (1.25-1.47)	NA	3.29 (2.63-4.12)	NA	2.76 (2.33-3.27)	NA
Taiwan	1.51 (1.30-1.75)	1.19 (1.01-1.39)	1.53 (1.32-1.78)	1.14 (0.98-1.33)	1.20 (1.12-1.29)	1.05 (0.98-1.12)	1.22 (1.03-1.45)	1.15 (0.95-1.39)	1.46 (1.23-1.73)	1.12 (0.93-1.34)
Bangladesh	1.73 (1.24-2.42)	NA	1.81 (1.27-2.59)	NA	1.12 (0.90-1.41)	NA	1.75 (1.08-2.83)	NA	1.59 (1.06-2.39)	NA
Philippines	NA	1.12 (0.97-1.31)	NA	1.13 (0.97-1.32)	NA	1.09 (1.00-1.18)	NA	0.97 (0.80-1.19)	NA	1.12 (0.96-1.32)
Vietnam	1.27 (0.99-1.63)	NA	1.31 (1.00-1.72)	NA	1.12 (0.95-1.31)	NA	1.16 (0.84-1.60)	NA	1.37 (1.02-1.84)	NA
Cyprus	1.23 (0.61-2.51)	1.05 (0.55-2.00)	1.14 (0.55-2.36)	1.06 (0.54-2.10)	0.85 (0.56-1.29)	1.07 (0.89-1.29)	1.40 (0.56-3.51)	1.05 (0.47-2.34)	1.15 (0.45-2.90)	1.06 (0.46-2.47)
Czech Republic	NA	1.38 (0.93-2.07)	NA	1.48 (0.97-2.26)	NA	1.50 (1.17-1.94)	NA	1.31 (0.67-2.57)	NA	NA
Estonia	1.09 (0.74-1.60)	1.30 (0.76-2.24)	1.10 (0.74-1.65)	1.30 (0.74-2.28)	0.99 (0.82-1.21)	1.13 (0.93-1.37)	1.09 (0.71-1.67)	1.52 (0.74-3.13)	1.17 (0.73-1.88)	1.21 (0.60-2.48)
Croatia	1.26 (0.62-2.55)	NA	1.14 (0.52-2.52)	NA	0.75 (0.49-1.14)	NA	1.47 (0.41-5.26)	NA	NA	NA
Hungary	1.95 (1.34-2.84)	1.62 (1.30-2.02)	1.87 (1.27-2.75)	1.59 (1.28-1.99)	0.98 (0.80-1.20)	1.12 (1.02-1.23)	1.99 (1.09-3.66)	1.28 (0.98-1.67)	2.10 (1.52-2.90)	1.65 (1.28-2.12)
Lithuania	1.07 (0.82-1.39)	1.52 (0.91-2.53)	1.06 (0.80-1.40)	1.40 (0.83-2.37)	0.99 (0.85-1.14)	1.07 (0.88-1.30)	1.14 (0.78-1.67)	3.27 (1.57-6.81)	1.11 (0.80-1.56)	1.54 (0.90-2.65)
Latvia	1.04 (0.77-1.39)	1.12 (0.53-2.34)	1.05 (0.78-1.43)	1.10 (0.50-2.42)	1.04 (0.88-1.21)	1.04 (0.82-1.32)	1.09 (0.78-1.52)	1.02 (0.37-2.82)	1.02 (0.71-1.45)	1.04 (0.43-2.53)
Poland	1.56 (1.01-2.40)	1.22 (0.91-1.63)	1.53 (0.99-2.37)	1.18 (0.89-1.58)	1.15 (0.91-1.44)	1.02 (0.91-1.14)	1.15 (0.72-1.85)	1.32 (0.92-1.89)	1.42 (0.82-2.46)	0.95 (0.59-1.51)
Romania	1.26 (0.70-2.29)	NA	1.20 (0.65-2.21)	NA	0.86 (0.61-1.22)	NA	1.68 (0.68-4.14)	NA	1.29 (0.69-2.42)	NA
Slovenia	1.04 (0.75-1.43)	1.10 (0.61-1.97)	1.03 (0.74-1.44)	1.10 (0.61-1.98)	0.92 (0.77-1.11)	1.19 (0.95-1.50)	1.12 (0.71-1.77)	1.02 (0.44-2.37)	1.20 (0.74-1.95)	1.19 (0.61-2.33)
Slovakia	1.27 (0.93-1.73)	1.42 (1.07-1.88)	1.33 (0.95-1.85)	1.47 (1.09-1.96)	0.95 (0.81-1.11)	1.13 (1.01-1.27)	1.18 (0.77-1.82)	1.50 (1.04-2.17)	1.15 (0.82-1.59)	1.45 (1.04-2.02)
Austria	1.05 (0.94-1.19)	2.03 (1.49-2.76)	1.08 (0.95-1.22)	2.05 (1.50-2.81)	0.92 (0.86-0.99)	1.49 (1.31-1.71)	1.14 (0.95-1.37)	1.99 (1.26-3.13)	1.06 (0.93-1.20)	2.09 (1.49-2.94)
Belgium	NA	1.12 (0.68-1.87)	NA	0.98 (0.57-1.68)	NA	0.90 (0.66-1.24)	NA	0.59 (0.28-1.27)	NA	1.31 (0.75-2.27)
Germany	1.09 (0.97-1.24)	1.67 (1.38-2.02)	1.07 (0.95-1.21)	1.66 (1.37-2.02)	0.90 (0.85-0.96)	1.09 (1.00-1.19)	1.22 (1.03-1.44)	2.06 (1.57-2.70)	1.13 (1.03-1.24)	1.63 (1.32-2.01)
Denmark	1.31 (0.93-1.83)	1.83 (1.23-2.72)	1.35 (0.95-1.92)	1.84 (1.22-2.77)	1.01 (0.86-1.18)	1.12 (0.98-1.29)	1.11 (0.74-1.68)	1.60 (0.99-2.59)	1.32 (0.92-1.90)	1.92 (1.23-2.99)
Greece	1.23 (0.63-2.37)	1.10 (0.59-2.05)	1.21 (0.61-2.42)	1.11 (0.58-2.10)	0.93 (0.65-1.32)	1.03 (0.85-1.26)	1.10 (0.41-2.96)	0.86 (0.37-1.99)	1.44 (0.57-3.63)	1.01 (0.42-2.44)
Spain	1.14 (0.83-1.56)	1.36 (1.01-1.82)	1.12 (0.81-1.56)	1.37 (1.01-1.86)	0.86 (0.73-1.03)	1.10 (0.98-1.24)	1.07 (0.66-1.73)	1.18 (0.85-1.64)	0.96 (0.65-1.41)	1.29 (0.90-1.85)
Finland	1.02 (0.88-1.19)	1.96 (0.99-3.90)	1.01 (0.86-1.19)	1.76 (0.88-3.55)	0.94 (0.86-1.02)	1.39 (1.06-1.82)	1.08 (0.88-1.33)	1.35 (0.51-3.56)	1.11 (0.77-1.60)	1.15 (0.52-2.54)
France	1.03 (0.70-1.50)	NA	1.05 (0.69-1.59)	NA	0.93 (0.75-1.15)	NA	1.37 (0.69-2.72)	NA	1.08 (0.68-1.72)	NA
Ireland	1.24 (0.79-1.95)	1.29 (0.87-1.91)	1.22 (0.77-1.94)	1.33 (0.89-1.98)	1.15 (0.92-1.45)	1.04 (0.91-1.19)	1.36 (0.74-2.51)	1.29 (0.80-2.09)	1.28 (0.73-2.26)	1.31 (0.83-2.06)
Iceland	1.09 (0.71-1.68)	1.12 (0.58-2.17)	1.11 (0.72-1.71)	1.13 (0.57-2.22)	1.00 (0.82-1.21)	1.12 (0.91-1.37)	1.13 (0.77-1.65)	1.05 (0.47-2.37)	1.05 (0.62-1.77)	1.02 (0.42-2.46)
Italy	1.17 (0.76-1.81)	1.19 (0.78-1.80)	1.22 (0.77-1.93)	1.20 (0.78-1.85)	1.05 (0.85-1.28)	1.05 (0.91-1.22)	1.53 (0.91-2.59)	1.07 (0.64-1.80)	NA	NA

Luxembourg	1.03 (0.57-1.86)	NA	1.04 (0.56-1.93)	NA	0.95 (0.65-1.40)	NA	1.43 (0.56-3.66)	NA	0.93 (0.49-1.77)	NA
Malta	1.25 (0.65-2.41)	1.21 (0.63-2.34)	1.19 (0.60-2.35)	1.16 (0.61-2.19)	0.86 (0.61-1.21)	1.13 (0.92-1.39)	1.46 (0.54-3.97)	1.06 (0.43-2.63)	1.67 (0.66-4.18)	1.30 (0.57-2.97)
Netherlands	1.08 (0.70-1.65)	NA	1.09 (0.70-1.70)	NA	0.86 (0.68-1.10)	NA	0.98 (0.48-1.99)	NA	1.11 (0.70-1.77)	NA
Norway	NA	1.89 (1.20-2.98)	NA	1.83 (1.15-2.90)	NA	1.33 (1.09-1.62)	NA	1.44 (0.73-2.86)	NA	1.59 (0.94-2.67)
Portugal	1.28 (0.70-2.36)	NA	1.32 (0.67-2.58)	NA	1.14 (0.75-1.72)	NA	1.24 (0.44-3.51)	NA	1.14 (0.50-2.61)	NA
Sweden	1.10 (0.77-1.59)	1.16 (0.73-1.83)	1.13 (0.77-1.64)	1.19 (0.74-1.91)	1.01 (0.88-1.18)	1.03 (0.88-1.19)	1.12 (0.79-1.61)	0.88 (0.51-1.51)	1.12 (0.76-1.65)	1.17 (0.70-1.95)
United Kingdom	1.14 (0.87-1.50)	1.15 (0.89-1.49)	1.12 (0.85-1.48)	1.17 (0.90-1.51)	1.04 (0.92-1.17)	1.06 (0.96-1.18)	1.18 (0.83-1.67)	0.97 (0.70-1.35)	1.14 (0.86-1.52)	1.17 (0.88-1.54)
USA	NA	1.16 (0.91-1.48)	NA	1.19 (0.92-1.53)	NA	1.06 (0.92-1.23)	NA	0.95 (0.67-1.34)	NA	1.15 (0.88-1.51)
Mexico	1.18 (0.95-1.46)	1.46 (1.28-1.67)	1.14 (0.91-1.42)	1.49 (1.30-1.69)	1.09 (0.96-1.22)	1.21 (1.14-1.28)	1.12 (0.82-1.52)	1.23 (1.04-1.46)	1.18 (0.89-1.56)	1.46 (1.27-1.68)
Brazil	1.87 (1.52-2.29)	1.17 (1.04-1.32)	1.83 (1.49-2.27)	1.19 (1.06-1.34)	1.52 (1.35-1.71)	1.00 (0.95-1.05)	2.05 (1.53-2.74)	1.03 (0.88-1.19)	1.87 (1.43-2.45)	1.17 (1.03-1.33)
Ecuador	1.14 (0.99-1.32)	1.02 (0.93-1.11)	1.14 (0.98-1.32)	1.02 (0.92-1.12)	1.06 (0.97-1.16)	1.05 (1.01-1.09)	1.25 (1.05-1.49)	0.97 (0.87-1.08)	1.14 (0.97-1.33)	1.02 (0.92-1.14)

Note: RR, relative risk; CIs, confidence intervals; NA, not available.

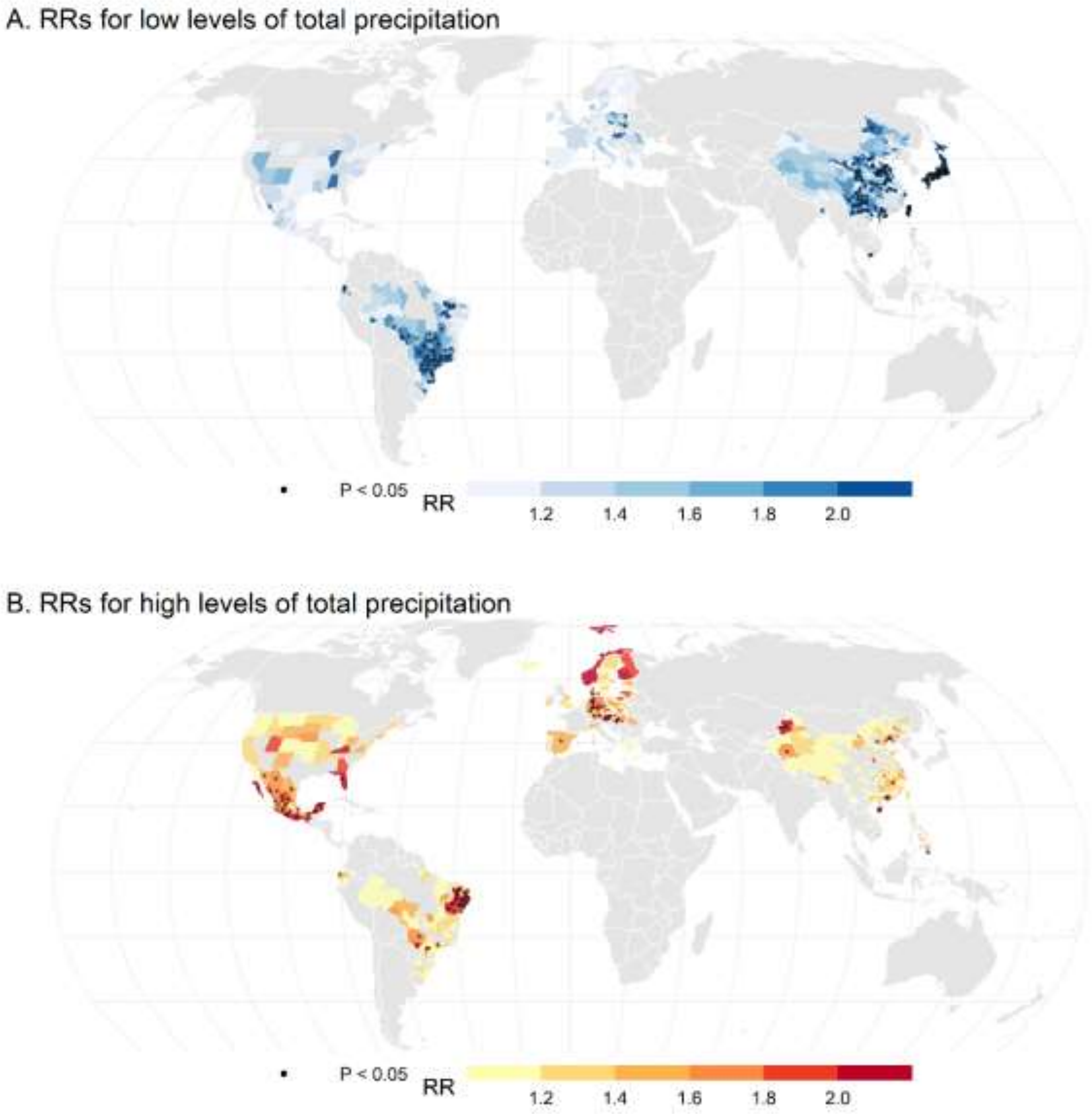

Figure S1. The location-specific RRs for low and high levels of total precipitation shown as spatial map. *RR, relative risk.*
